# A Web-Based Dyadic Intervention to Manage Psychoneurological Symptoms for Patients With Colorectal Cancer and Their Caregivers: Protocol for a Mixed Methods Study

**DOI:** 10.2196/48499

**Published:** 2023-06-28

**Authors:** Yufen Lin, Laura S Porter, Wonshik Chee, Olatunji B Alese, Kimberly A Curseen, Melinda K Higgins, Laurel Northouse, Canhua Xiao

**Affiliations:** 1 Nell Hodgson Woodruff School of Nursing Emory University Atlanta, GA United States; 2 Department of Psychiatry and Behavioral Sciences School of Medicine Duke University Durham, NC United States; 3 Winship Cancer Institute Emory University Atlanta, GA United States; 4 School of Nursing University of Michigan Ann Arbor, MI United States

**Keywords:** chemotherapy, colorectal cancer, intervention, symptoms, web-based program

## Abstract

**Background:**

Patients with colorectal cancer (CRC) receiving chemotherapy often experience psychoneurological symptoms (PNS; ie, fatigue, depression, anxiety, sleep disturbance, pain, and cognitive dysfunction) that negatively impact both patients’ and their caregivers’ health outcomes. Limited information is available on PNS management for CRC patient and caregiver dyads.

**Objective:**

The purposes of this study are to (1) develop a web-based dyadic intervention for patients with CRC receiving chemotherapy and their caregivers (CRCweb) and (2) evaluate the feasibility, acceptability, and preliminary effects of CRCweb among patient-caregiver dyads in a cancer clinic.

**Methods:**

A mixed methods approach will be used. Semistructured interviews among 8 dyads will be conducted to develop CRCweb. A single-group pre- and posttest clinical trial will be used to examine the feasibility, acceptability, and preliminary effects of the intervention (CRCweb) among 20 dyads. Study assessments will be conducted before (T1) and after intervention (T2). Content analysis will be performed for semistructured interviews. Descriptive statistics will be calculated separately for patients and caregivers, and pre-post paired *t* tests will be used to evaluate treatment effects.

**Results:**

This study was funded in November 2022. As of April 2023, we have obtained institutional review board approval and completed clinical trial registration and are currently recruiting patient-caregiver dyads in a cancer clinic. The study is expected to be completed in October 2024.

**Conclusions:**

Developing a web-based dyadic intervention holds great promise to reduce the PNS burden in patients with CRC receiving chemotherapy and their caregivers. The findings from this study will advance intervention development and implementation of symptom management and palliative care for patients with cancer and their caregivers.

**Trial Registration:**

ClinicalTrials.gov NCT05663203; https://clinicaltrials.gov/ct2/show/NCT05663203

**International Registered Report Identifier (IRRID):**

PRR1-10.2196/48499

## Introduction

Colorectal cancer (CRC) is the third most common cancer and the second leading cause of cancer-related deaths in the United States [1]. Patients with CRC receiving chemotherapy often experience severe and distressing psychoneurological symptoms (PNS) that include fatigue, depression, anxiety, sleep disturbance, pain, and cognitive dysfunction [2-5]. When these multiple co-occurring symptoms are undertreated, they negatively affect functional status, survival rates, and quality of life (QOL) of patients and decrease health outcomes (eg, QOL and mood) of their family caregivers (hereafter “caregivers,” defined as family members or significant others identified by the patients as their primary source of emotional and physical support) [6-8]. Current interventional studies in context of CRC are mainly focused on CRC screening and treatment regimens [9,10]. Limited information is available on symptom management for patients with CRC and their caregivers, which underscores the critical need to develop an innovative and effective intervention to manage PNS for patient and caregiver dyads.

Evidence has shown that PNS may share similar mechanisms, such as increases in proinflammatory responses or neurotransmitter alterations, which partially explains why they cluster together [11,12]. Our prior work shows that depression and fatigue are core symptoms within PNS networks and that PNS are highly interconnected with each other over time, suggesting that symptom management for these clustered symptoms may decrease multisymptom burden at the same time [13]. Moreover, addressing multiple symptoms simultaneously is cost-effective as shown in prior research [14,15]. Although interventions have been developed for managing single symptoms (eg, pain and fatigue) [16,17], little is known about multiple PNS management.

Among current interventional programs, psychosocial strategies that decrease stress and improve psychosocial adaptation for individuals have demonstrated the potential of alleviating PNS as a cluster [18-22]. One evidence-based psychosocial intervention, the FOCUS program (ie, Family involvement, Optimistic attitude, Coping effectiveness, Uncertainty reduction, Symptom management), has shown to improve multiple co-occurring symptoms including PNS for patients with heterogeneous cancer diagnoses (eg, breast cancer and advanced CRC) and their caregivers [23-25]. However, FOCUS has several notable limitations, including an in-person delivery format that is costly and time-consuming. It is also not specific to PNS management and does not target patients with CRC receiving chemotherapy.

In addition, patients with CRC are facing unequal access to palliative care and disparities in treatment [26]. Prior studies reported that patients with CRC on Medicare and living in rural areas were more likely to experience severe symptom burden and treatment disruptions compared with those on private insurance and living in urban areas [27,28]. A technology-based approach (eg, web program) using computers and mobile devices allows high flexibility and accessibility for disproportionately affected patients with CRC and caregivers, especially during and after COVID-19, and minimizes the cost of the intervention in hectic and costly cancer clinics [29,30]. Therefore, developing a web-based dyadic intervention holds great promise to reduce PNS burden for both patients and their caregivers as well as to advance intervention development and implementation in cancer palliative care and health equity.

To our knowledge, there is no current web-based dyadic intervention for PNS management among patients with CRC and their caregivers. Therefore, the aims of this study are to (1) develop a web-based dyadic intervention to manage PNS for patients with CRC receiving chemotherapy and their caregivers (CRCweb) and (2) evaluate the feasibility, acceptability, and preliminary effects of CRCweb among patient-caregiver dyads in a cancer clinic.

## Methods

### Conceptual Model

**Figure 1:**
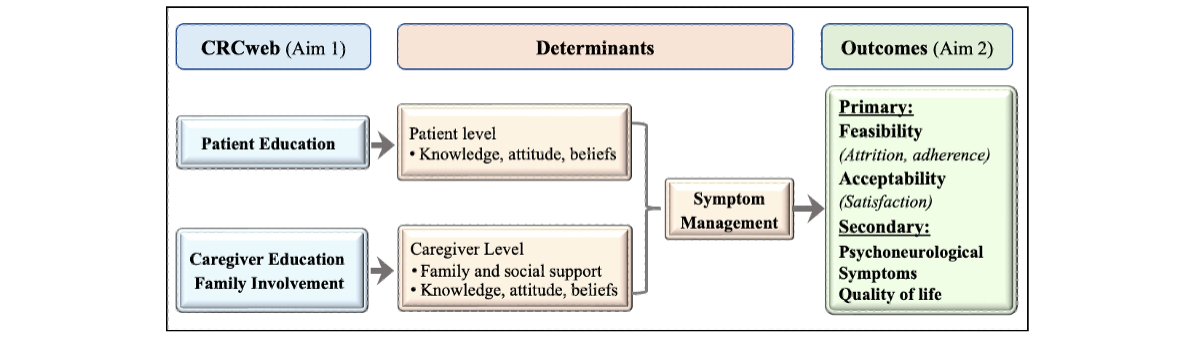
The web-based dyadic intervention model (CRCweb).

Leveraging the FOCUS program [23-25] and health education theory [31], we developed a web-based dyadic intervention model (Figure 1). The FOCUS program provides the rationale for psychosocial education (eg, coping strategies) [32] and family involvement and support by including family members in the care process and educating families to cope with multiple demands [33]. Health education theory provides the basis for enhancing the patient-caregiver communication process and working in team science [31]. We hypothesize that the CRCweb intervention will enhance PNS management by reducing symptom burden and increasing QOL through improving psychosocial education and family involvement and support for patients with CRC receiving chemotherapy and their caregivers.

### Design

The proposed study will be conducted in 2 phases—CRCweb development in phase 1 and a pilot clinical trial in phase 2. A mixed methods approach will be used. In phase 1, we will determine tailored content through qualitative interviews with patient-caregiver dyads, develop a web-based program prototype, and test program usability and refine the program to yield a final version of CRCweb. In phase 2, we will conduct a single-arm, pre- and posttest clinical trial to evaluate the feasibility, acceptability, and preliminary effects of the intervention on PNS and QOL among patient-caregiver dyads in a cancer clinic.

### Participants and Settings

In phase 1, we will recruit 8 patient-caregiver dyads; in phase 2, we will recruit 20 dyads. The inclusion criteria for patients will be age ≥18 years, diagnosed with CRC (any cancer stage), life expectancy >12 months, receiving active chemotherapy, self-reported at least 2 PNS (based on the symptom measures’ cutoff scores described in Table 1), identified primary caregiver, access to the internet, and fluent in English. Patients who have a Karnofsky Performance Scale score of <50 will be excluded. The inclusion criteria for caregivers will be age ≥18 years, primary caregiver, self-reported at least 2 PNS (based on the symptom measures’ cutoff scores described in Table 1), access to the internet, and fluent in English. Caregivers who have severe diseases such as cancer and heart disease will be excluded. Patients will be recruited at the Gastrointestinal Oncology Clinics of Emory Winship Cancer Institute.

**Table 1:** Measurements.

Outcome and variable				Time point^a^		Description
**Primary outcomes**						
	**Feasibility**					
		Attrition	T2		Attrition is measured by the number of patients and caregivers who drop out before completing the intervention. We will consider the intervention to have low attrition if >80% dyads remain enrolled.	
		Adherence	T2		Adherence is measured by the number of modules completed for dyads. It is determined by high adherence if dyads complete >80% of the modules.	
	**Acceptability**					
		Satisfaction	T2		Participant satisfaction with CRCweb is assessed by the 8-item Client Satisfaction Questionnaire (Cronbach α=.93; 8-item; 4-point Likert) among patients and caregivers at the end of the intervention.	
**Secondary outcomes^b^**						
	**Intervention effect**					
		Psychoneurological symptoms	T1, T2		Fatigue: 20-item Multidimensional Fatigue Inventory. A cutoff score of ≥43.5 indicates moderate-to-severe fatigue. Depression: 8-item Patient Health Questionnaire. A cutoff score of ≥10 indicates clinically significant depression. Anxiety: Spielberger State Anxiety Inventories. A cutoff score of >32.2 indicates high levels of state anxiety. Sleep disturbance: Pittsburgh Sleep Quality Index (PSQI). PSQI score of ≥5 indicates a significant level of sleep disturbance. Cognitive dysfunction: Attentional Function Index (AFI). AFI score of <5 indicates a significant level of cognitive dysfunction. Pain: Brief Pain Inventory. A cutoff score of ≥4 indicates moderate to severe pain.	
		Quality of life	T1, T2		The Short Form-12 consists of 12 questions about physical and mental health as well as overall health status. It is scored into 2 components: physical component score (PCS) and mental component score (MCS). Higher PCS and MCS scores indicate a better quality of life.	

^a^T1: before intervention, at baseline; T2: after intervention.

^b^The details for the measures can be found in Multimedia Appendix 1.

### Ethics Approval

The study was approved by the institutional review board at Emory University (STUDY00004750) and registered at the CliniclTrials.gov (NCT05663203). Participants will be recruited from May 2023 to August 2024 at the Emory Winship Cancer Institute. The research staff will screen eligible patients in the electronic health record system (Epic); they will then reach out to health care providers at the clinic who will confirm eligibility and suitability and give permission for the research staff to reach out to the patient-caregiver dyads to assess their interests. If the research staff find that both patients and caregivers are interested, they will explain the study to dyads and obtain dyads’ written informed consent in person at the clinic.

### Phase 1—CRCweb Development

#### Semistructured Interviews

We will conduct semistructured interviews with patient-caregiver dyads using an interview guide developed from the existing literature that includes open-ended questions and probes. Dyads (n=8) will be asked about their needs for the intervention, perspectives, and suggestions for a web-based intervention to manage their symptoms, what they like and do not like about the proposed CRCweb intervention components (ie, family involvement, symptom management, and coping effectiveness) and delivery mode (eg, doses and intervals), and what modifications they would recommend being made. The interview will last 30 to 45 minutes and be conducted by the principal investigator (PI) in a private conference room at the clinic or in a Zoom meeting based on the dyad’s preference. All interviews will be audio recorded and transcribed, and field notes will be taken to capture nonverbal expressions.

#### Development of a CRCweb Prototype

We will develop the CRCweb prototype and deploy it to Amazon Web Services (AWS) at Emory. AWS at Emory is a certified cloud service provided by AWS. A technology expert (WC) who has developed several rigorous web programs such as TCOLA (a technology-based CRC support program for Asian American women) will provide technical support. Two full-stack web designers will develop the CRCweb prototype (in 4-6 months). We will also collaborate with Health Information Technology Core at Emory University School of Nursing for the web program development. The research team will review the CRCweb prototype to provide feedback and make modifications.

#### Usability Testing

After the CRCweb prototype is developed, we will ask 4 patient-caregiver dyads to use the CRCweb prototype and provide feedback about the usability of the program [34]. Semistructured interviews (30-45 minutes) will probe for participants’ experiences with using CRCweb (eg, design, navigation, structure, and language). Interviews will be recorded and transcribed; field notes will capture dyads’ interactions with the program and document problems with using the websites. Five clinicians or researchers in oncology area will serve as content experts and provide feedback on the program. Feedback will guide the refinement of the CRCweb program, yielding a final version that will be used in the clinical trial.

### Phase 2—Pilot Clinical Trial

We will conduct a single-arm, nonrandomized, and nonblinded pre- and posttest clinical trial among patients with CRC receiving chemotherapy and their caregivers (n=20 dyads). Primary outcomes (feasibility and acceptability) will be assessed at the completion of intervention among patients and caregivers. Secondary outcomes (PNS and QOL) will be assessed at baseline (T1, before intervention) and 8 weeks (T2, after intervention) for patients and caregivers. We will collect quantitative assessment data on internet-accessible devices via REDCap self-administered surveys. We will avoid collecting symptom data at the acute symptoms stage (within 1 week after chemotherapy administration) to reduce measurement bias. Additionally, semistructured formative evaluation interviews will be conducted after the intervention among these participants regarding their experiences and suggestions on the intervention to inform the future refinement.

#### Intervention

**Figure 2:**
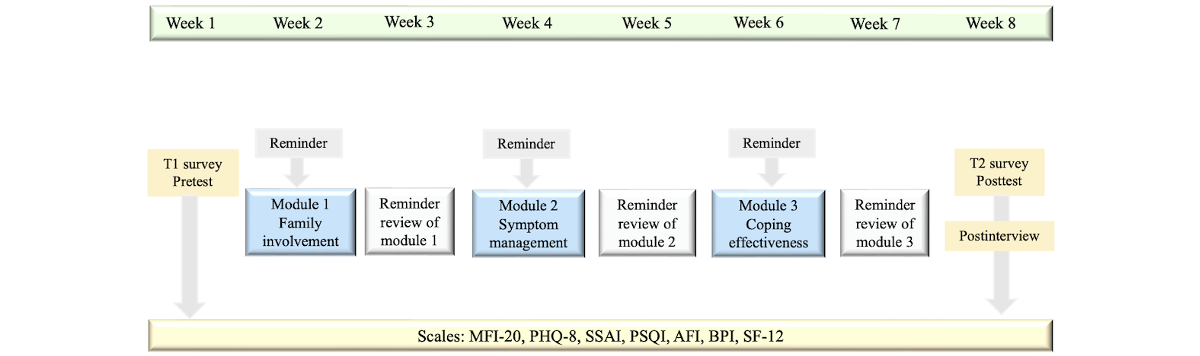
Flow of the intervention and assessments. AFI: Attentional Function Index; BPI: Brief Pain Inventory; MFI-20: 20-item Multidimensional Fatigue Inventory; PHQ-8: 8-item Patient Health Questionnaire; PSQI: Pittsburgh Sleep Quality Index; SF-12: Short Form-12; SSAI: Spielberger State Anxiety Inventories.

CRCweb is designed to create opportunities for patients and caregivers to obtain information and interact with each other. Patient-caregiver dyads (n=20) will participate together in an 8-week CRCweb intervention using assigned accounts and passwords to access to CRCweb with the help of the research staff. An introductory video (5 minutes) on the CRCweb page will teach patients and caregivers how to use CRCweb and navigate the websites. Dyads can use the program at any time and any place during the 8 weeks. They are encouraged to log on the program using their own devices and complete modules together, sitting side by side if possible. Three modules (ie, family involvement, symptom management, and coping strategies) are selected because they are more relevant to symptom management. Each module will be presented 2 weeks apart to enable dyads to absorb the information sequentially and practice the module’s principles before moving to the next module. The content of modules is shown in Table 2. Each module has 4 sections: video-enhanced lectures (10-20 minutes) and audio-enhanced texts, resources and web links, assignments, and evaluations. The video-enhanced lectures and audio-enhanced texts contain the same contents, allowing users to acquire the material according to their own learning styles. Modules include relevant resources and web links (eg, organizations, guidelines, and support services) for those who want additional information and resources. The modules for patients and caregivers are the same except tailored resources and web links. Assignments encourage dyads to practice learning skills and activities, discuss the symptoms and their effects on their daily lives, and brainstorm strategies to minimize the negative effects. After each module, a self-evaluation survey (score range 0-10) assesses the dyad’s learning. If they receive a score 0 to 5, a reminder message appears suggesting they take more time in the next week to review the module; if the score is 6 to 8, they can use the same amount of time next week to review the module; and if the score is 9 to 10, they are ready to move to the next module. We will track website activity data (eg, number of logins and time spent on the site) to assess if patients and caregivers engage in the learning modules. Additionally, we will send them weekly emails or SMS text messages to remind them to complete modules and surveys. The intervention flow is shown in Figure 2.

**Table 2:** Module content.

Module and goal			Content
**Family involvement**			
	Promoting open communication	•Discuss benefits of open communication versus concealment •Emphasize the importance of communication •Encourage open discussion of concerns •Use “I” statements to express feelings, use humor •Encourage sharing perspectives on any issues, concerns, or feelings •Address issues or concerns and reassure feelings	
	Encouraging mutual support and teamwork	•Discuss that cancer can affect both patient and family, making mutual support essential •Recognize the contributions of both members of the dyads and encourage the expression of appreciation	
	Identifying family strengths	•Help patients and family members identify individual and family strengths •Keep up positive outlook and face challenges together	
	Introducing family to information and resources	•Identify resources for the family to manage the recurrent illness •Give names and contact numbers of support groups for patients with colorectal cancer and their family	
**Symptom management**			
	Assessing symptoms	•Assess symptoms experienced by patients and their caregivers •Talk about the most severe, frequent, or distressed symptoms •Discuss effects of treatment on family relationships and the importance of caregiver’s health	
	Teaching self-care strategies	•Review patients’ and their caregivers’ symptom management and learn symptom management strategies •Provide information, guidelines, resources, and support services	
	Establishing realistic and short-term goals	•Set up easy goals to achieve for symptom management •Document and monitor symptoms, write symptom diary	
**Coping effectiveness**			
	Dealing with overwhelming stress	•Encourage day-to-day efforts to cope •Allow opportunities to discuss death and dying issues or concerns	
	Encouraging healthy coping and lifestyle behaviors	•Discuss benefits of active versus passive coping strategies •Educate about the importance of healthy lifestyle behaviors for patients and family members: eating and exercise, sleep and rest patterns, use of chemical substances, and support networks	
	Helping caregivers manage the demands of illness	•Encourage caregivers to accept offers of help from others •Help caregivers identify activities to restore their mental and physical energy (eg, hobbies and recreational activities)	

#### Intervention Fidelity

The PI will check CRCweb login information every Friday and send the reminder nudge messages with a checklist to patient-caregiver dyads who have not completed the modules and surveys. Given competing demands on patients and caregivers, we allow them to use CRCweb in their available time and limit the time of learning modules (less than 1 hour per week).

#### Measurement

A demographic questionnaire will be used to collect information such as age, gender, race or ethnicity, marital status, education, employment status, exercise, income, insurance, and dyadic relationships. Medical records will be reviewed for patients’ disease and treatment information, such as cancer sites, cancer stage (TNM, tumor-nodes-metastasis), treatment types, chemotherapy regimens and cycles, surgery, and radiotherapy. The measures that include primary outcomes (ie, attrition, adherence, and satisfaction) and secondary outcomes (ie, PNS and QOL) are shown in Table 1. As indicated, all the instruments are valid and reliable.

### Data Analysis

In phase 1, content analysis will be used to analyze the qualitative data [35,36]. Coding will be conducted by 2 researchers (including the PI) with backgrounds in cancer and qualitative methodology. The NVivo software (QSR International Pty Ltd) will be used to support data analysis, which is completed in four steps:

1. Data preparation [37]: 2 researchers read the transcripts together to get a sense of the whole picture; they identify key topics and storylines and categorize informational content (eg, patients’ perspectives).
2. Writing memos: using the transcripts and filed notes, 2 researchers write analytic memos and a brief case description of each participant.
3. Coding [38]: 2 cycles of coding will be used. The PI uses a priori codes from the literature and clinical practice to create a codebook. The first cycle includes codes developed a priori coding, descriptively, and in vivo [38]. The second researcher reviews the coding scheme and provides feedback; the PI revises the codebook by adding or combing codes. Once the first cycle of coding is completed, 2 researchers review the findings and develop larger categories of data using pattern coding and theoretical coding [38]. The second cycle will be used to organize and group codes that are similar or shared common characteristics.
4. Categorizing and connecting: 2 researchers construct tables and matrices to organize, condense, and display the codes and data samples into categories; all researchers help search for patterns, trends, and paradoxes across all participants and then summarize the descriptions and develop the themes [39].

In phase 2, quantitative data will be analyzed using SPSS Statistics (version 27.0; IBM Corp). Descriptive analyses will be conducted to determine the frequencies, percentages, means, and SDs of major variables. Feasibility of study attrition and adherence will be assessed by calculating attrition rates, identifying reasons for refusal, and assessing completeness of the data. Accessibility of participant satisfaction will be evaluated by calculating descriptive statistics to summarize responses to the poststudy evaluation items. Pre-post paired *t* tests (and nonparametric equivalents as needed) will be used to evaluate the changes between secondary outcomes (PNS, using the same calculating methods as described in our previous publication [13]) from pre- to postintervention stages for patients and caregivers. We will use dyadic analyses because of the increasing evidence that patients’ and their family caregivers’ responses to illness are interrelated [14]. Time and level (ie, patient vs caregiver) will be treated as within-subject variables to control for the interdependent nature of the data. The main effect by time will be examined to determine the overall effect of intervention on PNS for patient-caregiver dyads as a unit (average scores across dyads) [40]. We will also analyze time-by-level interactions to determine if there will be a differential effect of the intervention on patients’ and caregivers’ outcomes [14]. As needed (for an adjusted analysis), potential covariates (eg, age, insurance, and income) will be included in the planned models as appropriate. Additionally, qualitative data from postintervention interview will use the same analysis as in phase 1. Convergent parallel analysis will be used to compare and merge the quantitative and qualitative data when they have been completed [41]. We will discuss areas of convergence or divergence between the quantitative and qualitative results to refine the intervention.

## Results

Given that it is a study protocol, we do not yet have any results to report from the study. This study was funded in November 2022. As of April 2023, we have obtained institutional review board approval and completed clinical trial registration, and we are currently recruiting patient-caregiver dyads at a cancer clinic. The study is expected to be completed in October 2024. Expected results will be published in spring 2025.

## Discussion

To our knowledge, this study is the first to develop and evaluate a web-based dyadic intervention to manage PNS for patients with CRC during chemotherapy and their caregivers. The findings from this study will advance intervention development and inform implementation in cancer symptom management and palliative care.

There are several strengths that may enhance its impact. First, we develop and test an intervention to manage multiple co-occurring symptoms or symptom cluster (ie, a PNS cluster) for patients with CRC and their caregivers. Existing interventions have focused on managing single symptoms such as pain and fatigue, but interventions for a PNS cluster are still in early infancy [42]. Our program is designed to help improve PNS management for patients with CRC and their caregivers by providing dyads with information and support through educational sessions (eg, family support, symptom management, and coping) that are tailored to fit the needs of dyads.

Second, we will develop and evaluate a web-based intervention (CRCweb) that is accessible to patients and caregivers who may experience disparities in symptom management and palliative care. More than 76% of underserved populations have a smartphone or computer device that could provide access to web-based interventions [43]. Symptom management interventions have typically been delivered in person (eg, FOCUS), resulting in many barriers to participation (eg, long distance, limited provider availability, and high costs) [14]. In-person interventions can be problematic for patients and caregivers who tend to have many competing commitments and for providers who have limited time in clinic [44]. Web-based interventions have produced improvements in symptom management for patients with cancer and caregivers in several clinical trials [40,45-47], and the use of technologies and telemedicine has significantly increased during and after the COVID-19 pandemic [48]. A web-based dyadic intervention program may enhance feasibility and acceptability relative to in-person interventions by avoiding interruptions to treatments and reducing financial burden for patients and their families [49].

Third, we will evaluate the feasibility, accessibility, and preliminary effects of the intervention (CRCweb) to improve both patient and caregiver outcomes (eg, adherence, symptoms, and QOL). It is essential to focus on both patient and caregiver outcomes because caregivers experience increasing symptoms and distress while caring for patients [50]. To date, limited research has focused on symptom management and palliative care of both patients with CRC and caregivers. The proposed study will address the urgent need to develop a dyadic intervention to improve health outcomes for patient with CRC receiving chemotherapy and their caregivers and accelerate the development of palliative and psychosocial cancer care.

The study also has several potential pitfalls that should be considered. First, the study might have potential selection bias because the inclusion criteria are speaking English and the access to the internet using smartphones or computers. We estimated 10% to 20% of patients who may be interested in the study without mobile devices or internet access. We will provide them with smartphones and data plans. In addition, we will develop the web program in different languages in the future. Second, there might be attrition rate among participants due to treatment burden (eg, chemotherapy) or other issues. We developed several strategies for participant retention. (1) We planned a recruitment strategy that follows recommended guidelines [51] for recruiting and retaining seriously ill patients and underserved populations in longitudinal research and focuses on enrolling interested, completely informed participants. (2) We will train research staff to establish rapport from the time of initial contact and demonstrate rapport and flexibility throughout all study activities. (3) We will send reminder emails or SMS text messages to participants to complete intervention modules and surveys every week. (4) We will minimize participant burden through web-based data collection, limiting the number of measurements, and allowing flexibility in learning modules. (5) Participant concerns will be responded to immediately, within reason. (6) To promote retention between data collections, we will send “thank you” notes, birthday cards, and holiday greetings. (7) We will reimburse each dyad member for participating in the study (eg, US $25 per person for interview, US $50 per person for clinical trial using a prorated incentive plan that reimbursements are disbursed at weeks 1, 5, and 8).

The brevity of the intervention and delivery via web-based format enhance dissemination possibilities. Results from this proposed study may lead to advances in translation into clinical practice. For future research, we may develop a web-based multilevel interventions that include more levels (eg, clinician team, clinic, local, state, and nation level), conduct larger and multicenter interventions (eg, cluster randomized clinical trials), and implement the web-based intervention program in multiple clinical sites nationally and internationally (eg, effectiveness-implementation hybrid design). In addition, we will incorporate potential biomarkers along with interventions to further understand the effects of the interventions. The ultimate goals will be to develop the web-based dyadic intervention (CRCweb) program and disseminate and translate it into clinical settings. The website will become publicly available to equip patients and caregivers with tools to better manage their multiple co-occurring symptoms and improve QOL.

In conclusion, developing a web-based dyadic intervention holds great promise to reduce PNS burden and improve QOL for patients with CRC receiving chemotherapy and their caregivers. The findings from this study will advance intervention development and implementation in cancer symptom management and palliative and psychosocial care as well as accelerate the transformation of technology-based programs into clinical practice.

## Appendix

Multimedia Appendix 1 Measures.

Multimedia Appendix 2 Peer review report by Oncology Nursing Foundation Research Grants (USA).

## Abbreviations

AWS: Amazon Web Services
CRC: colorectal cancer
FOCUS: Family involvement, Optimistic attitude, Coping effectiveness, Uncertainty reduction, Symptom management
PI: principal investigator
PNS: psychoneurological symptoms
QOL: quality of life

## Data Availability

Data sharing is not applicable to this protocol paper as no data were generated or analyzed. The data set generated and analyzed in the interview and clinical trial will be shared with other users only under a data sharing agreement that provides a commitment to the following: (1) use of these data for research purposes only and not for the purposes of identifying individual participants, (2) the securing of the data and any copies (soft or hard) generated from these data using secure filing and appropriate secure computer technology, and (3) destroying or returning the data after analyses are completed. The PI (YL) will coordinate requests for data and maintain documentation for requests and distributions. Emory University has an established Institutional Data Use Agreement that can easily be adapted and deployed.
