# Supplementary material for: A Web-Based Dyadic Intervention to Manage Psychoneurological Symptoms for Patients With Colorectal Cancer and Their Caregivers: Protocol for a Mixed Methods Study
Source: JMIR Res Protoc. 2023 Jun 28;12:e48499. doi: 10.2196/48499 (PMC10365620; doi:10.2196/48499)
Supplement: Multimedia Appendix 1 [file resprot_v12i1e48499_app1.docx]

**Multimedia Appendix 1**

**Measures**

Depression is measured using the eight-item Patient Health Questionnaire (PHQ-8), a well-established valid and reliable self-administered diagnostic measure for depressive disorders in clinical studies (Pressler et al., 2011). The PHQ-8 asks the number of days in the past 2 weeks the respondent has experienced a variety of depressive symptoms. Each item is scored from 0 (not at all) to 3 (nearly every day); the sum of each item is the total score (0 to 24). Patients with a cutoff score of more than 10, clinically significant depression, will be referred to a clinical psychiatrist for further assistance (Kroenke et al., 2009).

Fatigue is measured using the Multidimensional Fatigue Inventory (MFI)-20. The MFI is a 20-item self-reported instrument that includes five dimensions of fatigue: general fatigue, physical fatigue, mental fatigue, reduced motivation, and reduced activity (Smets et al., 1995). Each dimension includes four items on a 1-5 scale. The total score, ranging from 20 to 100 (higher scores indicating more severe fatigue) is calculated as the sum of the five dimensions. The MFI-20 has well established validity and reliability (Schubert et al., 2007).

Sleep disturbance is assessed by a subjective measure, the Pittsburgh Sleep Quality Index (PSQI) (Buysse et al., 1989). Its validity and reliability have been established in various populations, including patients with cancer (Fontes et al., 2017). The PSQI consists of 19 items assessing sleep disturbances in seven dimensions (i.e., subjective sleep quality, sleep latency, sleep duration, habitual sleep efficiency, sleep disturbances, use of sleep medication, and daytime dysfunction). Each dimension scores from 0 (no difficulty) to 3 (severe difficulty), and the sum of these dimension scores is the global sleep quality (0 to 21). Higher scores indicate more difficulty in sleeping.

Cognitive dysfunction is assessed by the Attentional Function Index (AFI) (Cimprich et al., 2011). The AFI consists of 16 items designed to measure attentional function. A higher total mean score on a 0 to 10 indicates greater capacity to direct attention. The AFI score of < 5 indicates a significant level of cognitive dysfunction. The AFI has three subscales (i.e., effective action, attentional lapses, interpersonal effectiveness). The AFI has well established reliability and validity (Cimprich et al., 2011).

Pain is assessed by the Brief Pain Inventory (BPI) (Daut et al., 1983). Patients rate the intensity of the pain (i.e., now, average, worst) using 0 (none) to 10 (excruciating) numeric rating scales (NRS). They provide information on the length of time they are in pain, how often their pain occurs, locations of their pain, quality of the pain, pain’s level of interference with function, and their level of pain relief and satisfaction with pain treatment. A cutoff score of ≥4 indicates moderate-to-severe pain. The BPI has well established validity and reliability (Daut et al., 1983).

**References:**

Buysse, D. J., Reynolds, C. F., 3rd, Monk, T. H., Berman, S. R., & Kupfer, D. J. (1989). The Pittsburgh Sleep Quality Index: a new instrument for psychiatric practice and research. *Psychiatry Res*, *28*(2), 193-213. <https://doi.org/10.1016/0165-1781(89)90047-4>

Cimprich, B., So, H., Ronis, D. L., & Trask, C. (2005). Pre-treatment factors related to cognitive functioning in women newly diagnosed with breast cancer. *Psychooncology*, *14*(1), 70-78.

Cimprich, B., Visovatti, M., & Ronis, D. L. (2011). The Attentional Function Index--a self-report cognitive measure. *Psychooncology*, *20*(2), 194-202. <https://doi.org/10.1002/pon.1729>

Daut, R. L., Cleeland, C. S., & Flanery, R. C. (1983). Development of the Wisconsin Brief Pain Questionnaire to assess pain in cancer and other diseases. *Pain*, *17*, 197-210.

Fontes, F., Gonçalves, M., Maia, S., Pereira, S., Severo, M., & Lunet, N. (2017). Reliability and validity of the Pittsburgh Sleep Quality Index in breast cancer patients. *Support Care Cancer*, *25*(10), 3059-3066. <https://doi.org/10.1007/s00520-017-3713-9>

Kroenke, K., Strine, T. W., Spitzer, R. L., Williams, J. B., Berry, J. T., & Mokdad, A. H. (2009). The PHQ-8 as a measure of current depression in the general population. *J Affect Disord*, *114*(1-3), 163-173. <https://doi.org/10.1016/j.jad.2008.06.026>

Pressler, S. J., Subramanian, U., Perkins, S. M., Gradus-Pizlo, I., Kareken, D., Kim, J., Ding, Y., Sauvé, M. J., & Sloan, R. (2011). Measuring depressive symptoms in heart failure: validity and reliability of the patient health questionnaire-8. *Am J Crit Care*, *20*(2), 146-152. <https://doi.org/10.4037/ajcc2010931>

Schubert, C., Hong, S., Natarajan, L., Mills, P. J., & Dimsdale, J. E. (2007). The association between fatigue and inflammatory marker levels in cancer patients: a quantitative review. *Brain Behav Immun*, *21*(4), 413-427. <https://doi.org/10.1016/j.bbi.2006.11.004>

Smets, E. M., Garssen, B., Bonke, B., & De Haes, J. C. (1995). The Multidimensional Fatigue Inventory (MFI) psychometric qualities of an instrument to assess fatigue. *J Psychosom Res*, *39*(3), 315-325. <https://doi.org/10.1016/0022-3999(94)00125-o>
