# Supplementary material for: A Web-Based Dyadic Intervention to Manage Psychoneurological Symptoms for Patients With Colorectal Cancer and Their Caregivers: Protocol for a Mixed Methods Study
Source: JMIR Res Protoc. 2023 Jun 28;12:e48499. doi: 10.2196/48499 (PMC10365620; doi:10.2196/48499)
Supplement: Multimedia Appendix 2 [file resprot_v12i1e48499_app2.pdf]

ONCOLOGY NURSING FOUNDATION RESEARCH GRANTS

REVIEWER COMMENT-SCORING FORM

(Adapted from NIH Review Criteria)

Proposal #: **21**

Principal Investigator: **Yufen Lin**

Title of Proposal: **A web-based dyadic intervention to manage psychoneurological symptoms for patients with colorectal cancer and their caregivers**

Check One: ☒ **PRIMARY REVIEW** ☐ **SECONDARY REVIEW** ☐ **COLLATERAL REVIEW**

OVERALL IMPACT

Reviewers will provide an overall impact score to reflect their assessment of the likelihood for the project to exert a sustained, powerful influence on the research field(s) involved in consideration of the following five scored review criteria, and additional review criteria. An application does not need to be strong in all categories to be judged likely to have major scientific impact. *Impact Scoring Criteria scale can be found at the end of this form*

**Overall Impact** - After considering all of the review criteria, summarize the significant strengths and weaknesses of the application and state the likelihood of the project to exert a sustained powerful influence on the field. Be sure to provide sufficient information for the applicant to clearly understand the strengths and weaknesses that led to the overall score.	
**FINAL SCORE: 3**	
**Strengths**	• Intervention targets a prevalent and distressing PNS cluster in colorectal cancer patients and caregivers-innovation<br>• Web-based delivery is innovative and will enable wide reach and sustainability for future impact<br>• Experienced interdisciplinary team of researchers with necessary expertise (digital design, dyadic interventions, stats, recruitment, trials, symptoms)<br>• Research intensive environment with appropriate mentorship of PI-post-doctoral fellow (Lin) and supporting letters with clear articulation of support of fellow and contribution of team members<br>• Team has strong grant and publication track record<br>• Proposed study and gap in knowledge well-reasoned and guided by a systematic approach for adaptation phase that will support uptake and theoretical framework supporting intervention approach<br>• Lin has research skills in managing studies and a strong publication track record demonstrating productivity and an emerging focused program of research in symptoms and caregiver interventions<br>• Intervention builds on an evidence-based dyadic psychosocial intervention for PNS (FOCUS) with proven efficacy on outcomes<br>• Targets inclusion of racial and ethnic minorities in the adapting intervention phase.
**Weaknesses**	• Even though identifies purposeful sampling of racial/ethnic minorities-Inclusion criteria of English speaking and internet access may preclude participation of diverse population<br>• Definition of primary caregiver in the dyad not defined with potential for heterogeneity in outcomes (i.e. young daughter versus spouse)<br>• Measurement of outcomes in dyads-powered for patients and whether caregivers are symptomatic to meet inclusion criteria is not clear<br>• Mixed-method approach was not identified or referenced, nor the qualitative methodology component of mixed-methods defined;<br>• Lack of justification that web-based prototype can be developed in a 6-month timeframe and whether available across all digital formats and for $5000 and this could have been addressed in digital designer letter of support.<br>• How fidelity of the evidence-based FOCUS intervention will be addressed is not adequately described<br>• Analysis of between groups is this for pre-post or between caregivers and patients is not clear and confusing, nor how or what baseline variables will be controlled

- Unclear if metastatic colorectal cancer included or stage
-

SCORED REVIEW CRITERIA

Assigned reviewers will consider each of the five review criteria below in the determination of scientific and technical merit, and give a separate score for each.

1. Significance - Does the project address an important problem or a critical barrier to progress in the field? If the aims of the project are achieved, how will scientific knowledge, technical capability, and/or clinical practice be improved? How will successful completion of the aims change the concepts, methods, technologies, treatments, services, or preventative interventions that drive this field?

Score: 1

Strengths

- Proposed study addresses a prevalent and distressing problem and a gap in knowledge regarding PNS
- Use of an evidence-based intervention has potential for significant impact on health outcomes of colorectal cancer patients and their caregivers and reach given the use of web-based format.
-

Weaknesses *NONE Noted*

2. Investigator(s) - Are the PIs, collaborators, and other researchers well suited to the project? If Early Stage Investigators or New Investigators, or in the early stages of independent careers, do they have appropriate experience and training? If established, have they demonstrated an ongoing record of accomplishments that have advanced their field(s)? If the project is collaborative or multi-PD/PI, do the investigators have complementary and integrated expertise; are their leadership approach, governance and organizational structure appropriate for the project?

Score: 1

Strengths

- Strong interdisciplinary team with necessary expertise in skills required for the study, have established clinical and intervention expertise
- PI has skills in mixed-methods research with mentorship clearly identified and shows an emerging focused program of research with high publication track record.
- Organizational and infrastructure appropriate for the project.
- Consultant role of FOCUS intervention developer included will help to ensure appropriate adaptation and fidelity to evidence-based intervention.
-

Weaknesses **None Noted**

3. Innovation - Does the application challenge and seek to shift current research or clinical practice paradigms by utilizing novel theoretical concepts, approaches or methodologies, instrumentation, or interventions? Are the concepts, approaches or methodologies, instrumentation, or interventions novel to one field of research or novel in a broad sense? Is a refinement, improvement, or new application of theoretical concepts, approaches or methodologies, instrumentation, or interventions proposed?

Score: 1

Strengths

- Uses a systematic approach to adapt an evidence-based intervention for a PNS cluster to a web-based format to decrease barriers to access in colorectal cancer and caregivers
- Engagement of dyads in the web-based symptom and coping self-management of the PNS cluster in colorectal cancer is innovative and more dyadic interventions are needed
-

Weaknesses **None Noted**

4. Approach - Are the overall strategy, methodology, and analyses well-reasoned and appropriate to accomplish the specific aims of the project? Are potential problems, alternative strategies, and benchmarks for success presented? If the project is in the early stages of development, will the strategy establish feasibility and will particularly risky aspects be managed?

If the project involves clinical research, are the plans for 1) protection of human subjects from research risks, and 2) inclusion of minorities and members of both sexes/genders, as well as the inclusion of children, justified in terms of the scientific goals and research strategy proposed?

Score: 2

Strengths

- Justification for the study is well-reasoned and the aims are clearly articulated and aligned with the research methodology.
- Building on an evidence-based intervention
- Sample size appropriate for preliminary effectiveness and measures appropriate and psychometric soundness
- Content analysis approach well defined for qualitative data
-

Weaknesses

- Type of mixed-method design not identified or referenced
- How data sources will be mixed not specified nor methodology
- Definitions for caregivers not defined
- Justification for *m*oderate effect size across all proposed outcomes is weak (i.e. same for patients as caregivers?)
- Unclear how fidelity to the evidence-based FOCUS intervention will be assessed or addressed
- Qualitative methodology informing the interviews not defined for the study.
- Unclear how usability of the digital prototype will be usability tested prior to testing in clinical trial (short timeframe for prototype development and usability testing)
- No justification for 4 dyads as adequate for usability?
- Unclear if controlling for baseline PNS symptom scores or other variables in both patients and caregivers
-

5. Environment - Will the scientific environment in which the work will be done contribute to the probability of success? Are the institutional support, equipment and other physical resources available to the investigators adequate for the project proposed? Will the project benefit from unique features of the scientific environment, subject populations, or collaborative arrangements?

Score: 1

Strengths

- Research intensive environment and appropriate infrastructure for success.
- Letter of support attest to availability of resources and ability to recruit thee subject population.
- Project will benefit from consultation role of intervention developer (Northouse).
-

Weaknesses *None noted.*

-

ADDITIONAL REVIEW CRITERIA

As applicable for the project proposed, reviewers may consider the following additional items in the determination of scientific and technical merit, but will not give separate scores for these items.

Protections for Human Subjects –If applicable, did the application describe how informed consent will be obtained and the steps taken to protect participants' rights or the welfare of animals? Did the application identify any potential risks associated with participation in the project?

*(Comments **Required** if Not Appropriate is checked)*

☒ Appropriate ☐ Not Appropriate ☐ Not Applicable

Comments:

Data and Safety Monitoring Plan *(Comments **Required** if Not Appropriate is checked)*

☒ Appropriate ☐ Not Appropriate ☐ Not Applicable

Comments: describes data monitoring for completion, etc.

Data and Safety Monitoring Board (Applicable for Clinical Trials Only) *(Comments **Required** if Not Appropriate is checked)*

☐ Appropriate ☐ Not Appropriate ☒ Not Applicable

Comments:

Inclusion of Women, Minorities and Children - Applicable Only for Human Subjects Research – Did the application address the inclusion of women, minorities and children in developing a research design appropriate to the scientific objectives of the study. Inclusion is required unless a clear and compelling rationale shows that inclusion is inappropriate with the respect to the health of the subjects or that inclusion is inappropriate for the purpose of the study. Did the application provide information on the composition of the proposed study population in terms of sex/gender and racial/ethnic group and provide a rationale for selection of such subjects in terms of the scientific objectives and proposed study design.

*(Comments **Required** if Not Appropriate is checked)*

☒ Appropriate ☐ Not Appropriate ☐ Not Applicable

Comments: Explicit description of inclusion of racial and ethnic groups in study; although English speaking and Internet access required could limit generalizeability

Vertebrate Animals – Did the application address the involvement of live vertebrate animals as part of the scientific assessment according to the following five points: 1) proposed use of the animals, and species, strains, ages, sex, and numbers to be used; 2) justifications for the use of animals and for the appropriateness of the species and numbers proposed; 3) adequacy of veterinary care; 4) procedures for limiting discomfort, distress, pain and injury to that which is unavoidable in the conduct of scientifically sound research including the use of analgesic, anesthetic, and tranquilizing drugs and/or comfortable restraining devices; and 5) methods of euthanasia and reason for selection if not consistent with the AVMA Guidelines on Euthanasia.

*(Comments **Required** if Not Appropriate is checked)*

☐ Appropriate ☐ Not Appropriate ☒ Not Applicable

Comments:

The following consideration (Budget) is NOT part of the scientific review. No budget matters should be listed in the above scored criteria. Budget matters should NOT be discussed during the review until after final scoring has been completed. At that point the Chair will ask if there are any concerns about the budget.

Budget and Period of Support – Is the budget and the requested period of support are fully justified and reasonable in relation to the proposed research. For more details, please see *Budget Information.*

Recommended budget modifications or possible overlap identification *(Provide comments or state "Adequate")*

☒ Adequate

Comments: The Letter of support by the digital design co-investigator should have indicated feasibility of web program development for $5000 as this seems low-no reference to digital copy editing for digital format, etc. Payment for membership fees to professional societies (1000.00)?.

ADDITIONAL COMMENTS TO APPLICANT

Reviewers may provide guidance to the applicant or recommend against resubmission without fundamental revision.

Additional Comments to Applicant (Optional) – Please provide any additional guidance to the applicant or recommend against resubmission without fundamental revision.

-

ONCOLOGY NURSING FOUNDATION RESEARCH GRANTS

REVIEWER COMMENT-SCORING FORM

(Adapted from NIH Review Criteria)

Proposal #: **21**

Principal Investigator: **Yufen Lin**

Title of Proposal: **A web-based dyadic intervention to manage psychoneurological symptoms for patients with colorectal cancer and their caregivers**

Check One: ☐ **PRIMARY REVIEW** ☒ **SECONDARY REVIEW** ☐ **COLLATERAL REVIEW**

OVERALL IMPACT

Reviewers will provide an overall impact score to reflect their assessment of the likelihood for the project to exert a sustained, powerful influence on the research field(s) involved in consideration of the following five scored review criteria, and additional review criteria. An application does not need to be strong in all categories to be judged likely to have major scientific impact. *Impact Scoring Criteria scale can be found at the end of this form*

Overall Impact - After considering all of the review criteria, summarize the significant strengths and weaknesses of the application and state the likelihood of the project to exert a sustained powerful influence on the field. Be sure to provide sufficient information for the applicant to clearly understand the strengths and weaknesses that led to the overall score.

FINAL SCORE: 3

Strengths *(Please provide comments or state "None Noted")*

- Adaptation of an evidence-based intervention tested in almost 1000 participants
- Making an evidence-based intervention more accessible by moving it to an online format
- Including participants in adaptation of intervention

Weaknesses *(Please provide comments or state "None Noted")*

- It appears that only 3 of the modules of the in person intervention will be adapted – is there evidence that it will maintain its efficacy?
- Need further justification of a PNS symptom cluster – is there evidence for why creating a symptom cluster is superior?
- Intervention has never been used in CRC patients
- Need further clarification of why CRC caregivers experience possibly even more severe PNS symptoms than CRC patients

SCORED REVIEW CRITERIA

Assigned reviewers will consider each of the five review criteria below in the determination of scientific and technical merit, and give a separate score for each.

1. Significance - Does the project address an important problem or a critical barrier to progress in the field? If the aims of the project are achieved, how will scientific knowledge, technical capability, and/or clinical practice be improved? How will successful completion of the aims change the concepts, methods, technologies, treatments, services, or preventative interventions that drive this field?

Score: 3 *(Score Needed)*

Strengths *(Please provide comments or state "None Noted")*

- CRC is a prevalent disease with a high mortality rate
- Significant health disparities associated with CRC treatment and outcomes
- Recognition of dyadic role in CRC treatment

Weaknesses *(Please provide comments or state "None Noted")*

- Further information about FOCUS intervention would strengthen the application
- It appears that 3 modules of the FOCUS intervention will be adapted – is there evidence that this will maintain the intervention fidelity?
- Description of the intervention seems like coursework – slides with voiceover – further discussion of how loss of in person connection will impact adherence would strengthen

2. Investigator(s) - Are the PIs, collaborators, and other researchers well suited to the project? If Early Stage Investigators or New Investigators, or in the early stages of independent careers, do they have appropriate experience and training? If established, have they demonstrated an ongoing record of accomplishments that have advanced their field(s)? If the project is collaborative or multi-PD/PI, do the investigators have complementary and integrated expertise; are their leadership approach, governance and organizational structure appropriate for the project?

Score: 2 *(Score Needed)*

Strengths *(Please provide comments or state "None Noted")*
- Investigators appear to have appropriate expertise to conduct the proposed study

Weaknesses *(Please provide comments or state "None Noted")*
- None noted

3. Innovation - Does the application challenge and seek to shift current research or clinical practice paradigms by utilizing novel theoretical concepts, approaches or methodologies, instrumentation, or interventions? Are the concepts, approaches or methodologies, instrumentation, or interventions novel to one field of research or novel in a broad sense? Is a refinement, improvement, or new application of theoretical concepts, approaches or methodologies, instrumentation, or interventions proposed?

Score: 4 *(Score Needed)*

Strengths *(Please provide comments or state "None Noted")*
- Adaptation of an evidence-based intervention
- Web-based format for greater accessibility
- Use of intervention in a new population

Weaknesses *(Please provide comments or state "None Noted")*
- Adaptation plan is not clearly explained – it appears that the intervention will only be partially moved to an online format – need further explanation/justification
- Further explanation of how slides with accompanying voice over will engage participants as compared to in home nurse visits would strengthen

4. Approach - Are the overall strategy, methodology, and analyses well-reasoned and appropriate to accomplish the specific aims of the project? Are potential problems, alternative strategies, and benchmarks for success presented? If the project is in the early stages of development, will the strategy establish feasibility and will particularly risky aspects be managed?

If the project involves clinical research, are the plans for 1) protection of human subjects from research risks, and 2) inclusion of minorities and members of both sexes/genders, as well as the inclusion of children, justified in terms of the scientific goals and research strategy proposed?

Score: 3 *(Score Needed)*

Strengths *(Please provide comments or state "None Noted")*
- Mixed methods design
- Use of 8 dyads to inform adaptation
- Clear aims with concise outcome variables

Weaknesses *(Please provide comments or state "None Noted")*
- Additional discussion of the conduct of the qualitative portion and analysis plan would strengthen
- Additional discussion of the adaptation process of the intervention would strengthen

5. Environment - Will the scientific environment in which the work will be done contribute to the probability of success? Are the institutional support, equipment and other physical resources available to the investigators adequate for the project proposed? Will the project benefit from unique features of the scientific environment, subject populations, or collaborative arrangements?

Score: 1 *(Score Needed)*

Strengths *(Please provide comments or state "None Noted")*
- Applicants are at a strong research institution with a dedicated cancer center
- Applicants have demonstrated successful recruitment from planned enrollment site

Weaknesses *(Please provide comments or state "None Noted")*
- None noted

ADDITIONAL REVIEW CRITERIA

As applicable for the project proposed, reviewers may consider the following additional items in the determination of scientific and technical merit, but will not give separate scores for these items.

Protections for Human Subjects –If applicable, did the application describe how informed consent will be obtained and the steps taken to protect participants' rights or the welfare of animals? Did the application identify any potential risks associated with participation in the project?

*(Comments **Required** if Not Appropriate is checked)*

☒ Appropriate ☐ Not Appropriate ☐ Not Applicable

Comments:

Data and Safety Monitoring Plan *(Comments **Required** if Not Appropriate is checked)*

☒ Appropriate ☐ Not Appropriate ☐ Not Applicable

Comments:

Data and Safety Monitoring Board (Applicable for Clinical Trials Only) *(Comments **Required** if Not Appropriate is checked)*

☒ Appropriate ☐ Not Appropriate ☐ Not Applicable

Comments:

Inclusion of Women, Minorities and Children - Applicable Only for Human Subjects Research – Did the application address the inclusion of women, minorities and children in developing a research design appropriate to the scientific objectives of the study. Inclusion is required unless a clear and compelling rationale shows that inclusion is inappropriate with the respect to the health of the subjects or that inclusion is inappropriate for the purpose of the study. Did the application provide information on the composition of the proposed study population in terms of sex/gender and racial/ethnic group and provide a rationale for selection of such subjects in terms of the scientific objectives and proposed study design.

*(Comments **Required** if Not Appropriate is checked)*

☒ Appropriate ☐ Not Appropriate ☐ Not Applicable

Comments:

Vertebrate Animals – Did the application address the involvement of live vertebrate animals as part of the scientific assessment according to the following five points: 1) proposed use of the animals, and species, strains, ages, sex, and numbers to be used; 2) justifications for the use of animals and for the appropriateness of the species and numbers proposed; 3) adequacy of veterinary care; 4) procedures for limiting discomfort, distress, pain and injury to that which is unavoidable in the conduct of scientifically sound research including the use of analgesic, anesthetic, and tranquilizing drugs and/or comfortable restraining devices; and 5) methods of euthanasia and reason for selection if not consistent with the AVMA Guidelines on Euthanasia.

*(Comments **Required** if Not Appropriate is checked)*

☐ Appropriate ☐ Not Appropriate ☒ Not Applicable

Comments:

The following consideration (Budget) is NOT part of the scientific review. No budget matters should be listed in the above scored criteria. Budget matters should NOT be discussed during the review until after final scoring has been completed. At that point the Chair will ask if there are any concerns about the budget.

Budget and Period of Support – Is the budget and the requested period of support are fully justified and reasonable in relation to the proposed research. For more details, please see *Budget Information.*

Recommended budget modifications or possible overlap identification *(Provide comments or state "Adequate")*

☒ Adequate

Comments:

ADDITIONAL COMMENTS TO APPLICANT

Reviewers may provide guidance to the applicant or recommend against resubmission without fundamental revision.

Additional Comments to Applicant (Optional) – Please provide any additional guidance to the applicant or recommend against resubmission without fundamental revision.

-

ONCOLOGY NURSING FOUNDATION RESEARCH GRANTS

REVIEWER COMMENT-SCORING FORM

(Adapted from NIH Review Criteria)

Proposal #: **21**

Principal Investigator: **Yufen Lin**

Title of Proposal: **A web-based dyadic intervention to manage psychoneurological symptoms for patients with colorectal cancer and their caregivers**

Check One: ☐ **PRIMARY REVIEW** ☐ **SECONDARY REVIEW** ☒ **COLLATERAL REVIEW**

OVERALL IMPACT

Reviewers will provide an overall impact score to reflect their assessment of the likelihood for the project to exert a sustained, powerful influence on the research field(s) involved in consideration of the following five scored review criteria, and additional review criteria. An application does not need to be strong in all categories to be judged likely to have major scientific impact. *Impact Scoring Criteria scale can be found at the end of this form*

Overall Impact - After considering all of the review criteria, summarize the significant strengths and weaknesses of the application and state the likelihood of the project to exert a sustained powerful influence on the field. Be sure to provide sufficient information for the applicant to clearly understand the strengths and weaknesses that led to the overall score.

FINAL SCORE: 3

Strengths *(Please provide comments or state "None Noted")*

- The proposed study is exceptionally strong. The goal is to create a web-based intervention to manage psychoneurological symptoms for patients and families which is significant for the population of CRC. The proposal is well written and comprehensive. The aims are simple, measurable, attainable, targeted, and able to be accomplished within the time frame.

Weaknesses *(Please provide comments or state "None Noted")*

- Minimal weaknesses were identified and are of a minor nature..

SCORED REVIEW CRITERIA

Assigned reviewers will consider each of the five review criteria below in the determination of scientific and technical merit, and give a separate score for each.

1. Significance - Does the project address an important problem or a critical barrier to progress in the field? If the aims of the project are achieved, how will scientific knowledge, technical capability, and/or clinical practice be improved? How will successful completion of the aims change the concepts, methods, technologies, treatments, services, or preventative interventions that drive this field?

Score: 2 *(Score Needed)*

Strengths *(Please provide comments or state "None Noted")*

- Objective data were presented to support the creation of a web-based intervention for a pt/family member and to transform technology programs into clinical practice

Weaknesses *(Please provide comments or state "None Noted")*

- Less supported with evidence was how this program would "advance intervention development"

2. Investigator(s) - Are the PIs, collaborators, and other researchers well suited to the project? If Early Stage Investigators or New Investigators, or in the early stages of independent careers, do they have appropriate experience and training? If established, have they demonstrated an ongoing record of accomplishments that have advanced their field(s)? If the project is collaborative or multi-PD/PI, do the investigators have complementary and integrated expertise; are their leadership approach, governance and organizational structure appropriate for the project?

Score: 1 *(Score Needed)*

Strengths *(Please provide comments or state "None Noted")*

- The research team are seasoned investigators with experience with web-based interventions, creation of web-based programs, cancer treatment and QOL aspects of the family caregiver, and neurological symptom management.
- Drs. Xaio, Porter, Chee, Higgins, Curseen, and Northouse have extensive research experience as PIs, collaborators, and consultants. Research team does not appear to have overlapping expertise areas for this application. Several team members have prior experience working with this PI.

Weaknesses *(Please provide comments or state "None Noted")*

- None-noted

3. Innovation - Does the application challenge and seek to shift current research or clinical practice paradigms by utilizing novel theoretical concepts, approaches or methodologies, instrumentation, or interventions? Are the concepts, approaches or methodologies, instrumentation, or interventions novel to one field of research or novel in a broad sense? Is a refinement, improvement, or new a.pplication of theoretical concepts, approaches or methodologies, instrumentation, or interventions proposed?

Score: 1 *(Score Needed)*

Strengths *(Please provide comments or state "None Noted")*

- Web-based interventions focused on CRC are novel.

Weaknesses *(Please provide comments or state "None Noted")*

- None Noted

4. Approach - Are the overall strategy, methodology, and analyses well-reasoned and appropriate to accomplish the specific aims of the project? Are potential problems, alternative strategies, and benchmarks for success presented? If the project is in the early stages of development, will the strategy establish feasibility and will particularly risky aspects be managed?

If the project involves clinical research, are the plans for 1) protection of human subjects from research risks, and 2) inclusion of minorities and members of both sexes/genders, as well as the inclusion of children, justified in terms of the scientific goals and research strategy proposed?

Score: 3 *(Score Needed)*

Strengths *(Please provide comments or state "None Noted")*

- Strategies and methodology are appropriate for this application. A logical and thorough approach was presented with each aim and secondary aims. Prior experiences and alternatives to this proposal were presented.

Weaknesses *(Please provide comments or state "None Noted")*

- Absent from the aims is verification of symptoms, management, and evaluation from a team of experts after the web-based intervention is created.
- The generic outline of the topics, content, and targeted population was provided; however, more details are warranted to provide a comprehensive approach (content) to patient/family education.

5. Environment - Will the scientific environment in which the work will be done contribute to the probability of success? Are the institutional support, equipment and other physical resources available to the investigators adequate for the project proposed? Will the project benefit from unique features of the scientific environment, subject populations, or collaborative arrangements?

Score: 1 *(Score Needed)*

Strengths *(Please provide comments or state "None Noted")*

- Applicant proposes the study will be conducted in a comprehensive cancer center with a specialized supportive care clinic for the CRC patients. Authors provided some details on x`

Weaknesses *(Please provide comments or state "None Noted")*

- Few details on the patient/family dyad instructions at home to access the program. Did not described the minimum computer requirements to complete the program.

ADDITIONAL REVIEW CRITERIA

As applicable for the project proposed, reviewers may consider the following additional items in the determination of scientific and technical merit, but will not give separate scores for these items.

Protections for Human Subjects –If applicable, did the application describe how informed consent will be obtained and the steps taken to protect participants' rights or the welfare of animals? Did the application identify any potential risks associated with participation in the project?

*(Comments **Required** if Not Appropriate is checked)*

☒ Appropriate ☐ Not Appropriate ☐ Not Applicable

Comments: Proposal to be submitted to IRB

Data and Safety Monitoring Plan *(Comments **Required** if Not Appropriate is checked)*

☐ Appropriate ☒ Not Appropriate ☐ Not Applicable

Comments: Did not find a plan in the application

ONCOLOGY NURSING FOUNDATION RESEARCH GRANTS

REVIEWER COMMENT-SCORING FORM

Data and Safety Monitoring Board (Applicable for Clinical Trials Only) *(Comments **Required** if Not Appropriate is checked)*

☐ Appropriate ☒ Not Appropriate ☐ Not Applicable

Comments: No mention of a DSM Board

Inclusion of Women, Minorities and Children - Applicable Only for Human Subjects Research – Did the application address the inclusion of women, minorities and children in developing a research design appropriate to the scientific objectives of the study. Inclusion is required unless a clear and compelling rationale shows that inclusion is inappropriate with the respect to the health of the subjects or that inclusion is inappropriate for the purpose of the study. Did the application provide information on the composition of the proposed study population in terms of sex/gender and racial/ethnic group and provide a rationale for selection of such subjects in terms of the scientific objectives and proposed study design.

*(Comments **Required** if Not Appropriate is checked)*

☒ Appropriate ☐ Not Appropriate ☐ Not Applicable

Comments: Appropriately address all the inclusions.

Vertebrate Animals – Did the application address the involvement of live vertebrate animals as part of the scientific assessment according to the following five points: 1) proposed use of the animals, and species, strains, ages, sex, and numbers to be used; 2) justifications for the use of animals and for the appropriateness of the species and numbers proposed; 3) adequacy of veterinary care; 4) procedures for limiting discomfort, distress, pain and injury to that which is unavoidable in the conduct of scientifically sound research including the use of analgesic, anesthetic, and tranquilizing drugs and/or comfortable restraining devices; and 5) methods of euthanasia and reason for selection if not consistent with the AVMA Guidelines on Euthanasia.

*(Comments **Required** if Not Appropriate is checked)*

☐ Appropriate ☐ Not Appropriate ☒ Not Applicable

Comments:

The following consideration (Budget) is NOT part of the scientific review. No budget matters should be listed in the above scored criteria. Budget matters should NOT be discussed during the review until after final scoring has been completed. At that point the Chair will ask if there are any concerns about the budget.

Budget and Period of Support – Is the budget and the requested period of support are fully justified and reasonable in relation to the proposed research. For more details, please see *Budget Information.*

Recommended budget modifications or possible overlap identification *(Provide comments or state "Adequate")*

☒ Adequate

Comments:

ADDITIONAL COMMENTS TO APPLICANT

Reviewers may provide guidance to the applicant or recommend against resubmission without fundamental revision.

Additional Comments to Applicant (Optional) – Please provide any additional guidance to the applicant or recommend against resubmission without fundamental revision.

-

IMPACT SCORE CRITERIA			
**Impact**	**Score**	**Descriptor**	**Additional Guidance on Strengths/Weaknesses**
**High**	1	Exceptional	Exceptionally strong with essentially no weaknesses
	2	Outstanding	Extremely strong with negligible weaknesses
	3	Excellent	Very strong with only some minor weaknesses

**Medium**	4	Very Good	Strong but with numerous minor weaknesses
	5	Good	Strong but with at least one moderate weakness
	6	Satisfactory	Some strengths but also some moderate weaknesses
**Low**	7	Fair	Some strengths but with at least one major weakness
	8	Marginal	A few strengths and a few major weaknesses
	9	Poor	Very few strengths and numerous major weaknesses

Minor Weakness: An easily addressable weakness that does not substantially lessen impact
Moderate Weakness: A weakness that lessens impact
Major Weakness: A weakness that severely limits impact
